# Empowerment and pathologization: A case study in Norwegian mental health and substance abuse services

**DOI:** 10.1111/hex.12828

**Published:** 2018-09-03

**Authors:** Tone Larsen, Hildegunn Sagvaag

**Affiliations:** ^1^ Division of Psychiatry District General Hospital of Førde Førde Norway; ^2^ Faculty of Health and Social Sciences Western Norway University of Applied Sciences Bergen Norway; ^3^ University of Stavanger Stavanger Norway; ^4^ Departement of Public Health The Faculty of Health Sciences University of Stavanger Stavanger Norway

## Abstract

**Context:**

Service user involvement in service development and research is an international goal. However, research illuminating the patient stakeholder role is limited.

**Objective:**

The aim was to explore what may hinder patients’ voices being heard when collaborating with staff and leaders to improve services.

**Design:**

This action research project targeted Norwegian public mental health and substance abuse services, utilizing co‐operative inquiry principles. Data were collected and member‐checked collaboratively by the researcher and coresearchers.

**Results:**

Results centre on patient involvement in services, service development and research. The patient voice was regarded as important but not necessarily decisive, as patients’ change needs could be perceived as pathology‐based. Patients provided feedback about fellow patients and medication—opioid maintenance treatment, in particular. Barriers to patient involvement included patients not being permitted to influence other patients’ individual treatment and a leader's difficulty accepting patients’ medication advice. Additionally, an apparent hierarchy among the professionals may have disempowered some staff members.

**Discussion:**

Results point to an organizational diagnostic culture, where stigmatizing and risk pathologization may limit patient input. Empowerment appeared to be perceived as something allowed by the staff and leaders, at their discretion. Although all parties may have agreed that patient involvement was valuable, acting as a united group about opioid maintenance treatment appeared difficult.

**Conclusion:**

Barriers to patient involvement may hinder the availability and efficacy of patients’ perspectives in service development. Awareness about reciprocal empowerment might contribute to service users’ voices being heard, enabling a united voice from service users and providers regarding service development.

## 1 INTRODUCTION

The World Health Organization (WHO) lists service user involvement and making services more responsive to service users’ needs as key objectives in their Comprehensive Mental Health Action Plan 2013‐2020. There is a need for more research on service development that includes inputs from these stakeholders.^1^ Although prior research has shown that patient involvement is necessary in service development, there is limited research about the patient stakeholder role.^2^, ^3^, ^4^, ^5^, ^6^, ^7^, ^8^, ^9^ In Norway, “user participation in health and social services” has been a policy aim since 1988^10^ (p. 28); indeed, it was a prerequisite for upgrading public services in national action plans for mental health and substance abuse services.^11^, ^12^ However, user participation in these services has been evaluated as insufficient.^7^, ^13^ While service user participation in decision making has been explored,^14^, ^15^, ^16^ obstacles to patient voices in mental health and substance abuse service development remain under‐researched.

We conducted an action research project in Norwegian public specialized mental health and substance abuse services (SMHS) to explore service user participation in service development.^17^, ^18^, ^19^ The study's primary objective was to develop a “user participation method” that ensured both service user and service provider impact on service development—with the idea that the process of knowledge development would benefit from patient, staff and leader involvement and participation in the research^20^ and in turn could inform service development initiatives and decisions. In research, public *involvement* (when service users/patients/carers are involved in the design/delivery of the research) is often distinguished from *participation* (when data are collected from them in interviews or trials).^21^ However, in the context of services and service development, user/patient *involvement* (as a term) is not used in relevant Norwegian legislation.^22^, ^23^, ^24^, ^25^ In national guidelines and action plans, *involvement* and *participation* are used interchangeably.^7^, ^12^, ^26^ These two concepts are therefore distinguished in our research design but, in the rest of this article, *involvement* encompasses both participation and involvement in research, services and service development. With this approach, we investigate elements that appear to hinder patients’ impact on decision making and their ability to be heard. Drawing on theories of *empowerment* 27, 28, 29 and *pathologization*,^30^, ^31^, ^32^ we discuss obstacles to patient involvement and address the following question: What may keep patients’ voices from being heard in their collaboration with staff and leaders to improve mental health and substance abuse services?

### 1.1 Empowerment and pathologization

Emerson claims that power is relational. The power to control or influence someone “resides in control over the things he values”^33^ (p. 32): How much one invests in goals mediated by another—and whether those goals can be achieved elsewhere—determines how dependent one is on that person. If one person is power‐disadvantaged, *balancing operations* may be set in motion, restoring an unbalanced relation by increasing or decreasing dependence between the parties to reduce the power advantage.^33^ Empowerment thus springs from power and can be interpreted as a three‐term concept: strength **→** force **→** power^34^ (p. 21). As such, “persons or groups that are in a situation of disempowerment shall acquire the strength and power to emerge from disempowerment”^34^ (p. 21). One approach is for service providers to strengthen service users’ ability to gain control over their lives.^34^ Paradoxically, service providers may then assume that they are empower*ing*, rather than *collaborating with,* service users. So that, empowerment may be taken from service users and returned to them diluted, as a reproduction or magnification of oppressive practices. However, the potential for *self‐empowerment* among *both* service users and providers must be considered.^35^ Empowerment can be individual and reciprocal when service providers and service users engage in a joint cause—collaboration and active dialogue between the parties may thus be conducive to collective empowerment,^27^ as “self‐directing persons develop most fully through fully reciprocal relations with other self‐directing persons”^17^ (p. 3).

Freire regards oppression as a hindrance to one's pursuit of self‐affirmation and liberation.^27^ His liberationist philosophy is a founding part of empowerment theory,^27^ in which “learning to perceive social, political and economic contradictions, and to take action against the oppressive elements of reality”^27^ (p. 35) is a central tenet. Freire describes a process that makes people responsible subjects participating in history, encouraging them to pursue self‐affirmation.^27^ Although contextual knowledge and collective empowerment are fundamental to this theory, self‐empowerment is an integral component. Individuals must seize their own empowerment when “engaged in the fight for their own liberation”^27^ (p. 53). Freedom from oppression is thus “acquired by conquest, not by gift”^27^ (p. 47). When in a position to embrace their freedom, the oppressed can “unveil” and confront the culture of domination through transformational action and reflection—a process Freire terms *praxis*. He argues that the oppressed must commit to unveiling the world through praxis and that dialogue can collectively empower both parties to name and transform dominant structures together.^27^, ^28^, ^36^

Changing public services can be challenging in an organizational culture founded on professional traditions; these patterns of assumptions represent distinctive organizational cultures that recreate problem‐solving mechanisms, ensuring harmony and predictability.^37^ In 1965, Løchen described an organizational *diagnostic culture* that muted the impact from the collision of roles, ideals and systems in a Norwegian psychiatric hospital.^30^ In this diagnostic culture, causes of behaviour were attributed to the individual patient, hindering patients from promoting their claims because their protests could be added to existing pathological assumptions. Furthermore, patients had to be controlled because they “might use their right to codetermination in a way that is harmful to themselves or conflicts with the system”^30^ (p. 219). Current literature points to a contemporary diagnostic culture wherein life problems may similarly be perceived as pathological conditions or somatic diseases.^31^, ^32^ As such, *stigmatizing pathologization* is defined as moral judgement of “inappropriate” behaviour associated with certain diagnoses, and *risk pathologization* predicts hypothetical future scenarios using “a particular susceptibility to illness”^32^ (p. 286). This process can constitute discriminating stigmatization and enable self‐pathologization. *Depathologization* is described as an attempt to change what is viewed as incorrect pathologization of behaviours.^32^

## 2 CONTEXT

The inquiry was conducted in a voluntary inpatient SMHS treatment unit in Norway where opioid maintenance treatment (OMT) was part of the services. OMT is increasingly provided in Norway, with 7055 patients in treatment in 2013.^38^ It is an “interdisciplinary specialized treatment for opioid dependence where the requisitioning of addictive medicines in a specific dosage (substitution treatment) is one measure in the overall rehabilitation process”^39^ (§3). Methadone and buprenorphine (Subutex/Suboxone) aim to maintain or block opioid receptors in the brain, thereby preventing withdrawal and cravings for opioids.^40^ According to OMT legislation, SMHS is responsible for initiating and downscaling medication.^39^ However, national OMT guidelines state that the risks of relapse and overdose mortality are high,^40^ so termination should “not be recommended unless there is good reason to believe that the patient will manage without opioids”^40^ (p. 90).

### 2.1 Research design

This descriptive single‐case study applied the cyclical principles of action research, starting with conceptualizing and particularizing the problem and moving through several interventions and evaluations.^17^, ^18^, ^19^, ^41^, ^42^, ^43^, ^44^ The inquiry was designed in line with co‐operative inquiry principles^45^—researching *with* rather than *on* people seemed an appropriate way to facilitate patient, staff and leader collaboration on knowledge and service development.^46^, ^47^

### 2.2 Involvement and participation

This research design ensured that patient coresearchers (PCs), staff coresearchers (SCs), leader coresearchers (LCs) and the researcher (first author) could collaborate on developing interview guides, data collection, interpreting and disseminating findings, and proposing service changes. During the 3 years of research, a total of 109 (66 m, 43 f) consent forms were signed. Staff contributors consisted of *treatment* and *milieu* staff. In this article, both groups are defined as staff, with distinctions between the groups specified when necessary. A division between patients in OMT and other patients was made in the results.

All patients chose gift cards over cash payment as compensation for their contribution in work groups, training, interviews, dialogue seminars and disseminating findings outside the research context.^1^ Staff and leaders who contributed outside regular working hours were compensated with equivalent time off.

**Figure 1:**
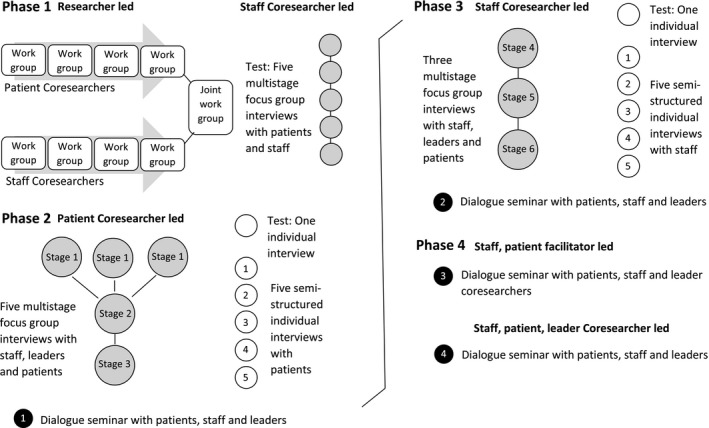
The four phases of inquiry

The researcher's motivation to initiate this project was anchored^48^ in her personal experience with addiction and outpatient mental health services, and her work as a social consultant in this SMHS. She facilitated the full inquiry while conducting participatory observation.^49^, ^50^ The researcher kept documentation in minutes and reports and made these accessible to the contributors. She attended all formal and most informal *service*‐related meetings that were relevant to the study's objectives, including treatment, staff, management meetings and the everyday morning meeting between staff, patients and (sometimes) leaders. She provided training and supervision to qualify coresearchers to lead test interviews and 6 stages of multistage focus group interviews^2^ ,^51^ 10 individual interviews^52^ and 4 dialogue seminars.^53^ In addition to service meetings and the *scheduled inquiry* (see Figure 1), there were ad hoc *inquiry* meetings with leaders, staff and/or patients to address any issues raised in the inquiry. For example, when staff and PCs encountered communication difficulties, a dialogue meeting was facilitated to clear up misunderstandings between staff and coresearchers in the inquiry. This meeting was neither scheduled inquiry nor service meeting, but was solely to encourage dialogue because of conflict.

**Table 1:** Participation and involvement in selected data

Scheduled inquiry and ad hoc inquiry meetings	Participation	Involvement/Coresearch
Before the inquiry One planning meeting (ad hoc)	Four leaders, one staff, one funder	
Phase 1One joint work group		Two patients, ten staff
Phase 2 One dialogue meeting (ad hoc)	Two leaders, two staff	Two patients
Individual interviews with patients	Patients 1, 3 and 4	One patient
Multistage focus group interviews		
Stage 1: one focus group	Three leaders	Two patients
Stage 1: one focus group	Four patients	Two patients
Stage 3: one focus group	Two patients, two staff, two leaders	Two patients
Phase 3 Individual interviews with staff	Staff 3 and 5	One staff

### 2.3 Four phases of inquiry

In continuous interplay between reflection, experience and action, practice was constantly refined.^46^ The project was structured according to the four phases of knowledge development in co‐operative inquiry: (a) propositional knowledge—knowledge expressed in theories or statements; (b) practical knowledge—that is, skills and competence; (c) experiential knowledge—knowledge developed in “direct encounter face‐to‐face with persons, places or things”^54^ (p. 230); and (d) critical scrutiny of the propositional knowledge—knowledge arising when the original propositions and questions are reconsidered and amended.^18^, ^45^, ^47^, ^3^ We used these phases as a framework only, as qualities from one phase may emerge (or merge with) another.^17^

*Phase 1:* The aim in this phase was agreement on a joint focus and to propose action.^20^ Here, it was important to explore and document coresearchers’ *propositional knowledge* about the SMHS.^18^ There were nine researcher‐led coresearch work groups—four with patients, four with staff. These SCs and PCs brainstormed about possible service developments, suggesting service improvements and training for staff and patients. They also developed interview guides for individual interviews with staff and patients and, in a final joint meeting, agreed on prioritized suggestions and established a joint focus: “to develop the services offered by this treatment facility for the better” (SCs and PCs, Joint Work Report). Their statements and propositions were documented in the Joint Work Report, which was used internally in the SMHS to inform plans and follow‐up actions. The report also provided a propositional knowledge baseline regarding the service developments that followed. Multistage focus group interviewing was assessed by SCs and the researcher as a potential method.

*Phase 2:* This phase's aim was to explore *practical knowledge*—that is how things were done.^46^ One PC conducted five semistructured interviews with patients. Of particular interest were the answers to the following questions: “Do you feel that what you say at the morning meeting is listened to?” “Do you think that it is difficult to express your opinion to staff and management for fear of consequences in your treatment?”

The interview guide for multistage focus group interviews was developed in this phase, with “What can be done to improve the treatment in the unit?” as the main question. The subquestions were: “What works well now and what could be improved? What works less well and what is the improvement potential for this? What is the treatment potential for occupational therapy, sports, trips, outdoor groups, music therapy? What is the treatment potential among patients themselves? What role should the staff take in the [treatment] setting?”

Three of the six multistage focus group stages (see Figure 1) were conducted in this phase. Two PCs led five multistage focus group interviews (data for this article are from three multistage focus group interviews in stages 1 and 3): three separate homogeneous group interviews with staff, leaders and patients (stage 1), followed by two heterogeneous interviews (stages 2‐3) with two participants from each group in stage 1 (excluding one leader who was replaced by another in stage 2). Each phase was finalized with a coresearcher‐led dialogue seminar^4^ where staff, leaders and patients were invited to explore service improvement potential. The suggested changes from all dialogue seminars were recorded in the Experience Report, including the individuals tasked with following them up. With highlights from the Joint Work Report also included, the Experience Report reflected the propositional knowledge acquired in each phase and informed plans and follow‐up actions, as experiences and service developments were recorded within the report.

*Phase 3:* The focus of this phase was on *experiential knowledge*, with the aim of elaborating and challenging assumptions and gaining creative insights.^46^ Here, superficial understandings from previous phases were explored as coresearchers deepened the inquiry. Two SCs conducted five semistructured interviews with staff. The answers to the following questions were of particular interest: “Do you feel that we have an overall vision to strive towards on the ward?” “Do you feel that you are able to make an impact as the primary contact person [for your patients]? Do you feel that you are heard (by the management/colleagues/the patients)?” “Does feedback from research influence practice?”

Together, the two SCs also led three heterogeneous multistage focus group interviews (stages 4‐6) and a dialogue seminar.

*Phase 4:* The aim of this final phase was to consider the *propositional knowledge* in the light of experiences from phases 1‐3.^20^ This required analysing former actions and the consideration of subsequent developments. A coresearch group of two leaders, two staff and three patients analysed an extract from the Experience Report about dialogue seminars 1 and 2. These coresearchers were supported by trained facilitators—one SC from phase 3 and the other a former patient—in a smaller dialogue seminar among themselves. These facilitators also supported the coresearchers in leading the final dialogue seminar, in which patients, staff and leaders participated.

### 2.4 Data collection

The minutes and reports were written in Norwegian and translated by a translation service. To ensure familiarity and readability, the researcher wrote in accordance with the documentation tradition in this SMHS. Therefore, these were predominantly condensed descriptions of the conversations, not verbatim transcriptions.^55^, ^56^, ^57^ Some interactions, however, were quoted word‐for‐word. To make a clear distinction between the two, verbatim sentences are underlined below. Relevant data for this article include the Joint Report, and minutes from a planning meeting, a dialogue meeting, five individual interviews and three multistage focus group interviews (see Table 1).

### 2.5 Data analysis

In this single‐case study, the relationship between researcher and contributors was “one of mutual and simultaneous influence”^58^ (p. 17). The minutes and reports were developed and interpreted in relationship between the contributors and researcher. Objectivity or generalization was not the intention. Rather, to ensure trustworthiness and authenticity, these data were rigorously *member‐checked* by the respective contributors, following Lincoln and Guba.^58^ The minutes/report were subjected to qualitative content analysis using NVivo 9.^55^, ^59^ Here, pathologization emerged as a central topic. The investigation alternated between induction and deduction, in qualitative *conventional* and *directed* content analysis.^59^ The results were interpreted in the light of empowerment^17^, ^27^, ^28^, ^29^ and pathologization literature.^31^, ^32^, ^60^, ^61^

The inquiry was approved by the Norwegian Centre for Research Data (NSD).

## 3 RESULTS

Our results indicate that although staff and patient empowerment were generally perceived as a goal, some leaders and staff were concerned that pathology could motivate—and therefore prejudice—patients’ contributions. Patients also reported having been and fearing being pathologized by staff. Further, our results point to the underlying assumption that patients were *allowed* to be involved.

### 3.1 “Change needs may be pathology‐based”

At the planning meeting, leaders and staff emphasized “the importance of offering satisfactory treatment” during the inquiry, urging the researcher to design the inquiry in a way that would “empower them [the staff] on par with the users” (planning meeting, leaders and staff). These participants were positive about patient involvement, but sceptical about letting patients make decisions:

It can be tempting in such a process to use pathological explanations to distinguish between desirable and undesirable behaviour. At the same time, the users’ change needs may be pathology‐based. It was emphasized in this context that the users’ voice is both relevant and legitimate in this project, but not necessarily decisive. (planning meeting, leaders and staff)

Patients also reported pathologization from staff. One perceived questions about how he slept as “a bit pathologizing, it's like cosseting adult people” (individual interview, patient 4), while others feared that staff misinterpretations could affect their diagnosis.

He is afraid of the consequences for his treatment if he says what he thinks here on the ward. … He fears that diagnoses are made based on something that the personnel have misunderstood and written about in his patient records. He has experienced that something said jokingly has been taken seriously… He finds that he needs to be careful what he says. (individual interview, patient 1)

Another patient explained how serious misinterpretations about him had been reported in his chart. He could not recognize the characteristics described—when he confronted the staff, the journal note was deleted. This experience “meant that his confidence to open up again to the personnel was undermined” (individual interview, patient 3):

The personnel must understand that their experience is not enough, they should know what they are talking about and the consequences of communicating a misunderstanding or making an incorrect report. … He questioned whether it is possible so early in the treatment to assign such big, burdensome labels, and that it needed to reach the point where he had to justify himself. The interview subject … believed that these characteristics might provide a basis for a future diagnosis if he had not told them otherwise. (individual interview, patient 3)

Thus, patients did report experiencing pathologization; some were careful with their words for fear of being misinterpreted and consequently pathologized.

### 3.2 “Permitted to be involved”

During the inquiry, staff and leaders changed attitudes towards patient involvement. Both staff and patients referred to decision making and involvement as something the patients “should be allowed to be part of” (individual interview, staff 5):

I think we have become much more conscious of being advised by patients, so that they are involved and allowed to be more involved in decisions about their treatment than previously. I think we have become much more aware of that … The interview subject confirms that she thinks the research process is constructive, as she finds user involvement to be something positive. Because as I said just now, the patients just had to go along with what was decided by the management and therapists. (individual interview, staff 3)

When it comes to user involvement and influencing their own treatment, the interview subject says that in a public system it is a matter of how far one is permitted to be involved. At the morning meeting, he has noted that occasionally he is given a hearing, but that issues raised can become stuck in the system and therefore feedback on patients’ questions may be inadequate. (individual interview, patient 1)

Some staff and patients doubted having an influence on treatment decisions:

Therapists and management have already decided … some matters have to be sent upwards before they come down again, in the sense that decisions must come from the therapists and the management, things cannot be resolved up front between the milieu staff and the patient. (individual interview, staff 5)

In general, he gets a hearing at the morning meeting if it is in the interest of the staff. It is possible to raise issues, but it has no impact. (individual interview, patient 4)

However, some patients did feel heard in the morning meetings. One patient said, “If he doesn't perceive that he is understood, he can take it up on a one‐to‐one basis” (individual interview, patient 3).

Some leaders set barriers to patient involvement: “The management emphasizes that user involvement should not extend to patients’ individual treatment, because the duty of confidentiality applies here” (leaders, stage 1). Concern for other patients was problematized by PCs in the dialogue meeting with leaders and staff:

A patient [PC] says that it is not permitted to raise such matters. You are cut off, you're not supposed to be frustrated about it. The patients [PCs] see this from a different perspective and believe that some people are being maltreated. The leader says it is important to have trust in the assessments that are made. But you cannot give an answer that is absolutely correct in such cases, and you are not allowed to inform the other patients about the case…They [PCs] understand that the staff want to help, but they have special insight after years of experience and knowledge of fellow patients’ situations. (PCs and leader, dialogue meeting)

In this meeting, the leader responded that it was difficult “to accept advice about medication, for example, because it can easily be manipulated” (leader, dialogue). The PCs, however, insisted on providing guidance because they “also have competence about how medicines work” (PCs, dialogue meeting).

Patients continued advising about medication, as they were concerned about large doses in OMT:

The interviewees feel very provoked to see patients [in OMT] who are on so much medication that you can see that they are high. … they [interviewees] do not fit in with Subutex treatment because they [patients in OMT] sit around sleeping/are stoned all day and night and do not take part in activities. Seeing them triggers the craving for drugs. (patients, stage 1)

What impression did patients who are dependent on lighter drugs get of the treatment when they saw OMT patients being allowed to get high at the state's expense? … He questioned the size of the doses and whether downscaling from Subutex was really a goal. (patient, stage 3)

One response to these concerns was that observing OMT patients could be regarded as something positive, in that it might discourage other patients: “The leader said that the experience is that OMT treatment is seen as attractive, while at the same time scaring people from going so far” (leader, stage 3). However, it seems the decision to provide OMT was never the leaders’ to make: “The requirement that all functions, including OMT, be covered is part of the Health Trust's plan for substance abuse” (leader, stage 3). Even so, the patients insisted on being heard about OMT beyond this context:

A participatory observer [PC] said it was important to stick to the realities, namely that addicts are prone to giving in to temptation. A patient urged following up this feedback, because change takes time. He also confirmed that this is a national drug and alcohol policy issue. He was supported on this point by the participatory observer [PC] and staff when he said that it is important that their experiences be listened to. (leader, PC, patient and staff, stage 3)

Some staff, patients and leaders seemed to agree that patient involvement was up to staff and leaders, but milieu staff explained that because treatment decisions were made on a higher hierarchical level, they were not allowed to make decisions in collaboration with the patients. Also, there were several distinct limitations to patient involvement.

## 4 DISCUSSION

Staff and leaders pointed to a need for empowerment among patients *and* staff, resonating with Freire's descriptions of dialogue between the oppressed and the oppressors to create change.^27^ However, in this context, balancing staff‐patient relations seemed challenging. Leaders and staff warned that it might be tempting to “stigmatize unwanted behaviour as pathological”^32^ (p. 281)—what Brinkman terms “stigmatizing pathologization”.^32^ Furthermore, predicting and preventing a scenario where pathology‐based suggestions influence decision making may be understood as risk pathologization.^32^ Patient empowerment seemed perceived as something that should be contextually controlled—a logic echoing the diagnostic culture where patients’ codetermination is controlled to prevent harm^30^: Thus, pathological assumptions on the individual level may have hindered patients’ ability to impact decision making on the systemic level.

Though patient involvement appeared to be a valued goal, by limiting patients’ impact on decision making the power‐advantaged may have ensured an imbalanced relation (following Emerson^33^). SMHS codes of conduct for staff and leaders stipulate that care of patients is their first concern, in the form of “satisfactory treatment”—a norm rooted in legal requirements, guidelines, organizational policy and patient expectations. Against this backdrop, perhaps staff and leaders believed that sometimes their own voice should carry more weight, and only so much empowerment was possible.

Many patients reported fear of becoming misunderstood and diagnosed incorrectly. One patient described confronting staff about certain characteristics in his chart that he felt were misattributed. His self‐justification may thus be understood as an attempt to depathologize himself. His reaction may also be interpreted as an act of self‐empowerment, as he freed himself from incorrect characteristics. This patient actively challenged “the dominant structure”^27^ and was arguably self‐empowered. One may also interpret this as an example of staff and leaders allowing empowerment; however, as Freire argues, empowerment is not a gift.^27^ Perhaps, a balancing operation enabled this power process, whereby the patient cultivated social relations with staff and leaders, thus acquiring the power advantage to influence their behaviour.^33^

Patients were increasingly invited to be involved, and status recognition may have influenced their involvement during the inquiry. Staff and leaders began facilitating patient involvement more often, which—together with patients’ experience of gratifications (via ego rewards and gift‐card compensation)—may have increased relational balance and encouraged patients’ involvement.^33^ However, both staff and patients also referred to patient decision making and involvement as something that the staff decided to allow. Thus, in this context, empowerment appears to have been perceived as something granted by the staff and leaders, rather than an opportunity seized by the patients.^27^ Furthermore, a staff‐patient relational imbalance also seemed apparent, due to a linear power network where staff intermediation between patients and decision makers was central.^33^ There also appeared to be a power imbalance between milieu staff, treatment staff and leaders. Interestingly, empowerment theory suggests that a disempowered staff may find it difficult to facilitate self‐empowerment among patients, potentially resulting in the replication and multiplication of oppressive practices.^35^ If the staff lacked experience with acting as responsible subjects participating in changing their own disempowered situation, how could they empower patients to take action against oppressive practices?

Other obstacles to patients’ voices being heard were apparent, including the leaders’ fear of manipulation and belief that patients should not be involved with fellow patients’ individual treatment. Additionally, several patients appeared committed to contributing their perspectives on OMT, but their arguments seemed obstructed by the leaders’ conviction that OMT deterrence was a positive. Freire's notion of praxis is useful in conceptualizing how these patients committed to unveiling, naming and transforming this situation.^27^ As such, the patients may have begun to peel back the veil on some deeply rooted dilemmas in mental health and substance abuse treatment.

Concurrent Norwegian studies suggest that these patients were not alone—findings demonstrate strong opinions about OMT among drug users, centred around the growing illegal spread of these drugs and OMT localization.^62^, ^63^ However, in our inquiry, certain OMT issues regulated by legislation, guidelines and the Health Trust seemed impervious to patients’ concerns: Although “high” patients were regarded as triggering to other patients, the SMHS was required to provide OMT; further, staff and leaders’ efforts to explore downscaling treatment may have been complicated by current guidelines stating that terminating OMT was not recommended; finally, managing OMT patient confidentiality in this (new) inquiry setting may have been challenging for staff and leaders. Consequently, these contextual barriers may have hindered staff and leaders from sharing reflections on OMT with patients. Emerson describes how balancing operations can unite a group in challenging surroundings,^33^ but it seems that although leaders, staff and patients agreed that patient involvement was valuable, acting as a united group—with one voice—around OMT was difficult.

## 5 CONCLUDING REMARKS

Results from our study of a Norwegian SMHS treatment unit target several barriers that may have hindered patients’ ability to be heard by staff and leaders: (a) feedback deemed by staff/leaders to be pathology‐based would not necessarily influence decision making; (b) patients were not permitted to impact fellow patients’ individual treatment; (c) empowerment seemed to be perceived as something to be controlled and granted by leaders and staff; and (d) due to contextual influences such as legislation, guidelines, organizational policy and codes of conduct, it may have been difficult for staff and leaders to listen to and explore patients advice.

We show how these barriers may limit the beneficial contribution of patients’ knowledge about more responsive services. Further, we sought to explore empowerment as something that can be genuinely reciprocal, irrespective of hierarchical positions or biology. A Freirean approach to empowerment among both staff *and* patients lies in contrast to controlling patients’ self‐empowerment. We suggest that reciprocal empowerment can potentially be enabled by facilitating self‐empowerment among service users and providers through training, supervision and explorative dialogue, centred on service user/provider awareness of (a) power dependence relations, and (b) barriers to and potential for service user/provider self‐ and reciprocal empowerment. This awareness might contribute to service users’ voices being heard, enabling a united voice from service users and providers around developing and transforming services.

One arena for further research is how reciprocal empowerment between service users and providers can be optimized when developing services. Potentially beneficial avenues for further inquiry include: (a) exploring how to optimize communication and the quality of individual and collective contributions in service development and (b) investigating which collaborative efforts either ensure or hinder sustained service quality improvement.

## CONFLICTS OF INTEREST

Division of Psychiatry, District General Hospital of Førde has been the lead author's employer. The author's interest has been in loyalty with the action research orientation.

## Notes

1 Excepting one former patient who received remuneration for collaborating with staff to provide drug training to staff and leaders.

2 Multistage focus group interviewing facilitates knowledge development about one topic through multiple stages; these occur predominantly with the same participants, using the same interview guide, to ensure deepening of perspectives and accumulated knowledge through explorative dialogue. A moderator encourages inquiry, supported by an observer who provides a summary of statements mid‐way through the interview and at the end of each interview for member feedback. A summary of the previous interview is presented in the next interview and participants are encouraged to continue the inquiry until ‘saturation’ is achieved. In this inquiry, there were *eight* multistep focus group interviews in six stages (excluding the five test multistep focus group interviews in phase one—see Figure 1): *five* multistep focus group interviews in phase two and *three* in phase three. Data for this article are from three of the five multistage focus group interviews conducted by patient coresearchers in phase two (see Figure 1 and descriptions below). The number of participants and coresearchers in these selected multistage focus group interviews is listed in Table 1, below.

3 Ideally, in a co‐operative inquiry, the same inquiry group would engage in all four phases. Here, however, different stakeholders’ perspectives dominated each phase—in the final phase, a co‐research collaboration between the stakeholders was achieved.

4 Dialogue seminars comprised the following: Coresearchers presented preliminary findings and proposed changes. Participants were asked to individually prioritize five topics (from the previous presentation or their own initiative) that they wanted to pursue in further dialogue about service improvement. Participants presented their topics in heterogeneous work groups, explaining their importance. The groups discussed the topics that emerged, agreeing on three categories to present in a final plenum discussion about the need for improvement. Finally, participants presented their categories and were encouraged by coresearchers to initiate and follow up with the suggested changes. (Data for this article were not collected from the dialogue seminars.).
